# Content Creators Between Platform Control and User Autonomy: The Role of Algorithms and Revenue Sharing

**DOI:** 10.1007/s12599-023-00808-9

**Published:** 2023-05-24

**Authors:** Tatjana Hödl, Thomas Myrach

**Affiliations:** Institute of Information Systems, University of Bern, Engehaldenstrasse 8, 3012 Bern, Switzerland

**Keywords:** Digital platform, Tension, Paradox, Algorithmic control, Perceived autonomy, Revenue sharing

## Abstract

Content creators generate and upload content on social media platforms. If these platforms have a revenue-sharing policy, content creators earn income from advertising revenue. This income is heavily dependent on the distribution of the content and the resulting view counts. Platform owners may exert algorithmic control that impacts content distribution, advertising income, and, consequently, the behaviour of content creators. The objectives of the platform owners combined with the interests of the content creators may lead to paradoxical tensions between the aims of control and autonomy. The opaque nature of algorithms coupled with the need to be recognised by the algorithm further reinforces this phenomenon. This study follows an interpretive qualitative research approach applying grounded theory methodology. This research uses semi-structured interviews with content creators to develop a theory explaining the tension between control and autonomy on revenue-sharing social media platforms. The study shows that algorithmic control and incentivisation create paradoxical tensions that affect the autonomy of content creators. Content creators attempt to minimise tensions of algorithm versus audience, regularity versus scheduling autonomy, and analytics versus decision-making autonomy in two ways: through self-centred measures such as improving metrics, pre-production, and being a pioneer and extraneous measures involving their own businesses, products, and sponsorships. This study sheds some light on the phenomenon of paradoxical tensions and provides guidance and strategies for content creators and platform owners about proceeding with their relationship. This study’s findings provide platform owners and decision-makers with a deeper understanding of the behaviour of content creators and the hurdles they face in platform work. The findings help them identify challenges, draw conclusions, and implement changes.

**Supplementary Information:**

The online version contains supplementary material available at 10.1007/s12599-023-00808-9.

## Introduction

Digital platforms demonstrate a powerful instrument for interacting with stakeholders (Bonina et al. 2021). Depending on the purpose of these platforms, the interactions may differ markedly. They include diverse functions such as electronic markets, job distribution, idea generation, and content publication. Platforms are also a vehicle for users to earn money by trading goods, offering services, and creating ideas and content. A growing number of people no longer engage in traditional employment relationships but work in flexible jobs as independent contractors and freelancers (Möhlmann et al. 2021). For them, digital platforms present an opportunity to adopt this way of life by generating money.

Earning money is clearly evident on online labour platforms such as Upwork, TaskRabbit, M-Turk, and Uber, because work is exchanged for money. By contrast, social media platforms utilise user-generated content, which is essentially published for free. Users have several potential sources of income, including revenue sharing by platforms (Cutolo and Kenney 2021). Revenue-sharing social media platforms allow users to generate income on digital platforms by providing content and receiving payment from advertising revenues. This is done through partner programs and incentive systems on platforms such as YouTube (Tang et al. 2012).

A fundamental component of digital platforms is sophisticated algorithms (Bonina et al. 2021). People who use digital platforms are increasingly exposed to algorithmic decision-making (Cram and Wiener 2020) because platforms rely on machine-learning algorithms to filter and control uploaded content (Agrawal et al. 2018; Curchod et al. 2020; Faraj et al. 2018). This activity includes incentive systems launched by platform owners. The algorithms decide which content achieves the targets set by the incentives and what payment content creators receive. It is increasingly difficult to comprehend sophisticated algorithms due to their complexity (Faraj et al. 2018). This is particularly problematic for content creators because they need to be recognised by the algorithms (Gillespie 2017) to distribute their content and generate income. This need may entice content creators to ‘play the visibility game’ (Cotter 2019, p. 895) by adapting to changes in the algorithms. Consequently, exposure to algorithmic control and incentivisation may cause tensions for content creators because they feel limited in their autonomy (Mazmanian et al. 2013; Putnam et al. 2014).

Current research has focused mainly on gig workers and developers, known as complementors, (Gawer 2021). Content creators entirely resemble neither gig workers nor complementors. Their purpose is to create content such as text, photos, and videos, which is more complex than the work of gig workers but less difficult than developing complementors’ software components. In a way, content creators are between the two streams of literature, which have so far neglected to consider content creation explicitly. Similarly to gig workers, content creators are subject to algorithms controlling their contribution. Within the platforms’ guidelines (e.g. YouTube: Community guidelines. https://www.youtube.com/howyoutubeworks/policies/community-guidelines/. Accessed 13 May 2022), content creators are autonomous in deciding what user-generated content they want to present. However, content is certainly rewarded or punished by incentivisation (Caplan and Gillespie 2020), which may limit content creators’ autonomy. How content creators experience algorithmic control and incentivisation and whether they feel restricted in their autonomy and adapt their behaviour accordingly is as yet poorly understood. Considering that more people are expected to work on digital platforms in the next few years (European Commission 2018; Mäntymäki et al. 2019), this phenomenon needs to be studied in more detail to understand the impact of digital platforms and algorithms on people, their well-being, and the broader welfare of society (Mirbabaie et al. 2022; Spiekermann et al. 2022).

This work addresses three research questions:

*How do the algorithms of social media platforms influence content creators’ behaviour?*
*How does revenue sharing on social media platforms influence content creators’ behaviour?*
*a) How do content creators adapt to the algorithm adjustments of social media platforms to ensure a reliable income?*
*b) How do content creators adapt to the revenue-sharing adjustments of social media platforms to ensure a reliable income?*

To answer these questions, we conducted an interpretive qualitative study (Orlikowski and Baroudi 1991) that uses grounded theory methodology (Charmaz 2014) to explore the view of content creators. We gained insights into the tension between the perceived control of the platform and the autonomy of the content creators with in-depth, semi-structured interviews with 26 content creators on revenue-sharing social media platforms such as YouTube and Twitch.

## Conceptual Background

Here, we introduce the context and outline the concepts relevant to our study. These concepts are derived from related literature. It should be noted that our findings followed grounded theory (Charmaz 2014) by emerging from the data; the concepts were not imposed on the data. However, to narrow the research problem and determine the methodological approach, we conducted a preliminary literature review, and when theoretical concepts appeared relevant, we integrated them with existing literature and theories (Urquhart and Fernández 2016, p. 141).

### Business Model of Social Media Platforms

Digital platforms are characterised by the fact that they mediate through technology, promote interactions between user groups, and allow user groups to perform certain tasks (Bonina et al. 2021). Social media platforms are digital platforms assigned to the transaction platforms because, as intermediaries, they enable information and content to be exchanged among users (Bonina et al. 2021; Gawer 2021). Social media platforms also belong to the category of multisided platforms because their business model serves several stakeholders (Cennamo and Santalo 2013; Evans and Schmalensee 2016; Veit et al. 2014). The most essential stakeholders are the platform owners themselves. Their platforms offer some valuable services to user groups. Users are another critical stakeholder group. They use the platform services for various purposes. Social media platforms emerged as networking sites among users to share content. Because the use of the platforms is usually free, advertising is a predominant element of the platform owners’ business model (Alaimo et al. 2020). Therefore, advertisers are another important stakeholder group. They want to present their promotional messages to the platform users directly or indirectly. Thus, the platform owners’ business model has to cohere with the interests of the two main stakeholder groups: it enables users to share content with each other, and it matches users with the advertisers’ promotion (Bonina et al. 2021).

In principle, all the users of a social media platform may provide content, which is therefore defined as user-generated content. However, certain users tend to be more active in generating content. In contrast, others tend to passively consume content created by others. The active users put considerable effort into producing content such as text, photos, and videos and are referred to as content creators (Albuquerque et al. 2012). Content creators are also called social media influencers (Freberg et al. 2011; Lou and Yuan 2019), opinion leaders (Casaló et al. 2020), and contributors (Tang et al. 2012). They all regularly post content on social media platforms and have a follower base with which they interact (Leung et al. 2022). Conversely, the passive users primarily consume content or interact minimally with likes and comments and are referred to as users (Albuquerque et al. 2012). Users are also referred to as followers (Leung et al. 2022) and subscribers (Tang et al. 2012) when they track the activity of certain content creators. Of course, these two types of use are not mutually exclusive, and one may expect content creators to consume other people’s content to a certain extent. Compared to traditional media, where for example, TV stations buy the content, the content on social media platforms is essentially provided for free. Therefore, the role of content creators is more complicated. Content creators want to deliver content that meets their interests, whether for attention, self-marketing, or monetary reasons (Tang et al. 2012), and this may be considered their business model. Content creators can be regarded as platform-dependent entrepreneurs (Cutolo and Kenney 2021). Thus, social media platforms may be considered multisided platforms that mainly involve three target customers: content creators, mostly passive users, and advertisers (Veisdal 2020).

The goals of content creators need not align with the interests of the platform owners or the advertisers. These latter groups are interested in attractive content that appeals to many users and attracts them to the platform. Therefore, platforms use algorithms and revenue sharing as control instruments to distribute content to the users and thus align the content to their business model and the commercial interests of the advertisers. The algorithms influence how successful particular content will be. This is important for content creators. Knowing the nature of the algorithm is crucial for them to align their content with the platform owners’ interests.

By distinguishing two user groups, we follow a broader definition of platforms and consider not only platform owners, developers, and end-users but also other stakeholders, such as advertisers and content creators, as part of the platform ecosystem (Kapoor et al. 2021; Qiu et al. 2017; Steininger et al. 2022). These independent groups interact and thus generate value (Rong et al. 2018). The content creators and users generate value by content creators’ ensuring that new content is always available and by users’ watching this content. This user base, in turn, is very attractive for advertisers (Evans and Schmalensee 2016).

### Management of Social Media Platforms

As described above, social media platforms bring together various stakeholder groups, each representing their own interests. Platform owners pursue their own and their advertisers’ interests through the management of social media platforms. Platforms use algorithms and in some cases revenue sharing to control content creators’ behaviour. This exposes content creators to perceived control. We disaggregate perceived control into algorithmic control and monetary control. We also outline content creators’ perceived autonomy and their need to be responsible for their own actions. Finally, we examine tensions that can arise between perceived control and perceived autonomy of content creators.

#### Perceived Control

##### Algorithmic Control

Research to date has mainly been concerned with the control of human controllers over human controlees, neglecting the role of algorithms in managerial control (Wiener et al. 2021). Because digital platforms are mediated through technology (Bonina et al. 2021), algorithms are an essential part of organisational control and transform it (Kellogg et al. 2020). Recent developments have shown that organisations also use algorithms to support managerial control and automate control processes (Wiener et al. 2021). Algorithms provide managerial support by helping the human controller to monitor and exercise control. Algorithms are also used instead of human controllers, sometimes known as algorithmic control (Wiener et al. 2021). Algorithmic control of platforms is used to control the behaviour of users on platforms to achieve the platform owners’ corporate objectives (Cram et al. 2020; Kellogg et al. 2020; Möhlmann et al. 2021). Instead of managers, algorithmic control is executed by the algorithm and displayed by a technology interface such as a smartphone app (Cram et al. 2020). All user groups are increasingly exposed to digital sensors and algorithmic decision-making (Cram and Wiener 2020) as platforms rely increasingly on machine-learning algorithms for control (Agrawal et al. 2018; Curchod et al. 2020; Faraj et al. 2018).

The effect of algorithmic control of social media platforms may be characterised as both direct and indirect. The direct effect of algorithmic control is the distribution of content on the platform. Content is presented to the users according to their anticipated interests. The distribution of content is communicated to its creators through the number of views. Not all views are counted and charged to advertisers. For example, on YouTube, the advertisements must be watched for at least 30 s, or the whole commercial if it is shorter (Pashkevich et al. 2012). Any advertising that users skip will not be charged and will not represent paid advertising views for content creators (YouTube Help: How engagement metrics are counted. https://support.google.com/youtube/answer/2991785?hl=en. Accessed 13 May 2022). The indirect effect of algorithmic control is the impact of content distribution on the behaviour of the content creators, who may adapt their content to reach more users. This is supported by some instruments on the platform. A feature of algorithmic control is that social media platforms collect masses of data that they display to content creators as performance metrics, such as the number of views, video ratings, and the number of subscribers (Qiu et al. 2015). It is important to note that this control happens only after completion and not during video creation. As soon as a video is uploaded and available on the platform, algorithmic control intervenes. Only then do content creators receive feedback through performance metrics. Content creators cannot bypass algorithmic control and ask questions about it because there is no room for negotiation. They can only adjust their behaviour for subsequent video content by anticipating how algorithmic control will manifest itself next time around. This form of control is comparable to output control (Kirsch 1997) because it transmits up-to-date information about their videos to the content creators. In output control, the ‘desired goals are articulated’ (Kirsch 1997, p. 217), and controlees are rewarded or sanctioned depending on whether the goals are achieved or not (Wiener et al. 2016). But in contrast to output control, social media platforms do not communicate concrete goals but merely refer to ‘focusing on what the audience likes’ (YouTube Help: Video discovery tips. https://support.google.com/youtube/answer/11914225?hl=en&ref_topic=11912225. Accessed 4 Nov 2022). This means the content that corresponds to the interests of the viewers is promoted because it is watched frequently and for a long time. However, the users’ interests do not have to coincide with those of platform owners or advertisers. In 2017 and 2018, incidents occurred of advertisements being shown alongside extremist content, causing advertisers to withdraw their engagement (Martinson 2017; Murphy et al. 2018). Platforms must therefore satisfy several interests. They not only weigh the interests of users but also distribute content that is conducive to the interests, policies, and goals of platform owners and advertisers. This brings us to our original argument: several groups including users, content creators, platform owners, and advertisers meet on platforms, each with their own interests and business models, and jointly generate value for the ecosystem.

##### Monetary Control

Some social media platforms use revenue sharing in addition to algorithmic control. Incentivisation is a common means of mitigating the principal–agent problem (Arthur and Jelf 1999; Atkinson et al. 1988; Black and Lynch 2004; Mortimer 2008) by aligning the actions of the agents with the goals of the principals. Freelancing content creators on social media platforms are not precisely comparable to employed agents, but the principal–agent problem is not limited to employment relationships. Directing content creators in the desired way remains critical to platforms, which is how incentivisation becomes a strategic instrument to foster the quality of the content (Tang et al. 2012). Monetary incentives seem to increase the number of stock recommendations (Chen et al. 2019) but reduce content contribution in online review communities (Sun et al. 2017). Two explanations for these contrasting findings seem to be motivation being crowded out and competition being crowded out. Motivation crowding out is the undermining of nonmonetary motivation, which leads content creators to stop contributing. Competition crowding out is increased competition, which leads content creators with low effectiveness to stop contributing (Liu and Feng 2021). Competition crowding out was found by YouTube when they introduced revenue sharing: user-generated content slowly declined while professionally generated content from media companies increased (Kim 2012). Social media platforms also use monetary incentives to exercise control and influence content creators’ behaviour. For example, YouTube uses different levels of compensation, ranging from ‘demonetised’ to ‘advertiser friendly’. The former is a punishment, and the latter is the most rewarding (Caplan and Gillespie 2020, p. 4). If a video is demonetised, it is still available and can be watched, but it has violated YouTube’s content policies for inappropriate language, violence, or adult content (YouTube Help: Advertiser-friendly content guidelines. https://support.google.com/youtube/answer/6162278. Accessed 2 Dec 2020), and a demonetised video does not generate advertising revenue. The opposite of demonetisation is when content is deemed advertiser friendly. The content complies with YouTube’s content policies and is approved for advertising revenue (Caplan and Gillespie 2020). It is unclear whether advertiser-friendly content is displayed more frequently. However, due to YouTube’s business model, this cannot be excluded, and its interest certainly lies in attaching advertising to as much content as possible. Further, content creators are exposed to dynamic pricing, expressed by the cost per thousand impressions or the cost per mille (CPM), a negotiated fixed price that advertisers are willing to pay every thousand instances of their advertisement being displayed to potential viewers, commonly known as impressions (Asdemir et al. 2012; Choi et al. 2020). It is in the nature of CPM that it changes and is determined by time, place, and display (Choi et al. 2020). Although incentivisation is often used, previous studies have not consistently shown that the quality of content changes as a result of revenue sharing (Chen et al. 2019; Liu and Feng 2021).

#### Perceived Autonomy

Autonomy is a prominent construct from psychology (Weber et al. 2020) and is, for instance, found in the self-determination theory (Ryan and Deci 2000) and the job characteristics model (Hackman and Oldham 1975). The self-determination theory plays a role in motivational research, with autonomy being one of the three psychological needs that increase self-motivation and improve well-being (Ryan and Deci 2000). In the job characteristics model, job autonomy describes a job’s freedom, independence, and the discretion an employee has in planning and executing their activities (Hackman and Oldham 1975).

Job autonomy can be understood as a multidimensional construct (Weber et al. 2020) which is composed of three dimensions: scheduling autonomy, including scheduling, planning, and prioritising of tasks; decision-making autonomy, using personal initiative or judgment to make decisions; and work methods autonomy, choosing procedures and methods for performing tasks (Breaugh 1999; Morgeson and Humphrey 2006; Ye and Kankanhalli 2018). We use Ye and Kankanhalli’s (2018, p. 166) definition of design autonomy and define perceived autonomy as ‘the extent to which individuals perceive that the platform allows them freedom and discretion to schedule work, make decisions, and choose methods for’ their content creation.

On social media platforms, content creators may achieve various aspects of autonomy. They have scheduling autonomy because they may plan and create content independently. For example, the platform does not specify when or how often they should upload posts. Content creators may also have decision-making autonomy if the platform does not restrict certain content. For example, YouTube has community guidelines that regulate sensitive and violent content. To enforce the community guidelines, YouTube relies on human reviewers and machine learning (YouTube: Community guidelines. https://www.youtube.com/howyoutubeworks/policies/community-guidelines/. Accessed 13 May 2022). Finally, content creators may have work methods autonomy if they specify the equipment, such as studio, camera, sound, and image resolution, entirely themselves.

#### Tensions Between Control and Autonomy

Content creators are subject to the influences of algorithmic control of the platform. However, they strive for autonomy in the generation and uploading of their content. These two may conflict, thus leading to tensions.

Tension is a term used to describe an emotional state that is evident when actors are stressed, feel uncomfortable, or behave anxiously when making decisions in organisational contexts. Scholars often use it as a superordinate construct that includes all paradoxical dynamics (Putnam et al. 2016). Tensions emerge when competing objectives collide and have the potential to conflict with each other (Lewis 2000; Smith and Lewis 2011). Both platform and tension literature acknowledge control and autonomy as a paradox (Mini and Widjaja 2019). In contrast to dilemmas, which involve two competing elements that each bear advantages and disadvantages (Smith and Lewis 2011), paradoxes arise when two opposing elements are fundamentally linked to each other and coexist (Smith and Lewis 2011).

The platform literature describes the paradox between control and autonomy (Mini and Widjaja 2019) as follows: Controllers influence the behaviour of controlees to achieve desired goals (Goldbach et al. 2018); conversely, autonomous individuals feel able to manage their planning, decision-making, and work methods despite the control (Ye and Kankanhalli 2018). In the case of content creators on social media platforms, the opposing elements are the algorithmic control and incentivisation of content distribution and the desire for autonomy over the generation and publication of content.

Paradoxes have been described as being managed by three response strategies: First, ‘either-or’ approaches consider the elements of the paradox to be independent of each other and try to resolve the tension by favouring one element over the other (Putnam et al. 2016). Second, ‘both-and’ responses express themselves by not favouring one extreme over the other but by switching between the poles or trying to achieve a balance between the two (Putnam et al. 2016). Third, with ‘more-than’ approaches, tensions are embraced to grow beyond them and thus create new opportunities (Putnam et al. 2016).

## Research Design and Methodology

### Design and Scope

To answer our research questions, we have chosen grounded theory methodology (Charmaz 2014) and considered the fundamental characteristics of various grounded theory streams (Birks et al. 2013). How content creators adapt to adjustments by the algorithms and what actions secure a reliable income is still unknown. The interpretive qualitative research approach (Orlikowski and Baroudi 1991) allowed us to gain further insights into the mechanics of platform incentivisation and how content creators perceived these mechanics. Because we want to explore the mechanics of incentivisation, we focus exclusively on revenue-sharing social media platforms (Tang et al. 2012). At the time of our study, only the video platform YouTube and the streaming platforms Twitch and Facebook Gaming used incentivisation. We mainly recruited content creators from YouTube because this was the dominant platform of the three (Suciu 2021). Two interviewees used both YouTube and Twitch, giving us some insight into the latter.

### Data Collection

To ensure that our findings are reliable and differentiated, we defined stereotypic personas for different types of content creators. The seven personas are based on real content creators we found during the first phase of recruiting interview participants. Three of the seven personas are described in Table 1. (in Online) Appendix A (available via http://link.springer.com) contains an overview of all personas. The content creators mentioned in Table 1 and Appendix A were not among the interview participants, but they inspired the personas and their names.

**Table 1:**
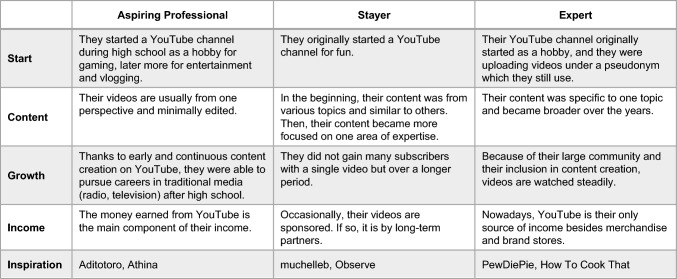
Examples of stereotypic personas of content creators and Appendix A: Personas of content creators

After defining personas, we assigned them to a growth-share matrix in Table 2. The two dimensions of our matrix were impact, measured by subscriber count, and involvement, measured by upload frequency. The dimension of impact was justified by the fact that there are small and large content creators, and we wanted to cover possible group differences. The dimension of involvement distinguishes between very active and less active content creators who make a larger or smaller contribution to the platform. When recruiting the interviewees, our matrix guided our selection, and we recruited content creators in line with the personas.

**Table 2:**
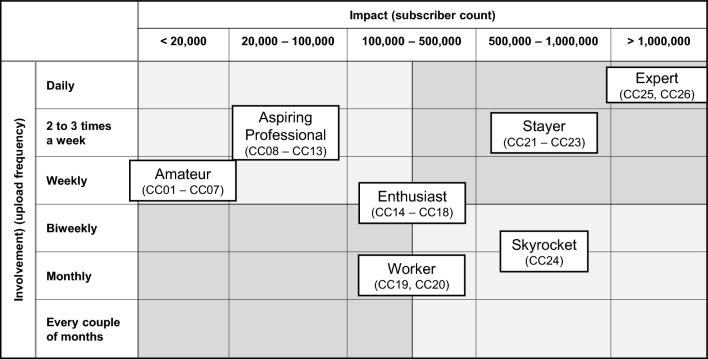
Involvement–impact matrix of content creators

To find possible interview partners, we started our search with the content creators whom we know through our own activity on the platform. We also used YouTube’s search function and search terms from popular content creation topics such as cooking and travelling. We used ordinary search engines to search for rankings and news articles that mentioned accounts of content creators. Some content creators had linked additional channels to their YouTube profiles that lead to either their own channels or other content creators. This led to a snowball effect and helped us considerably to bypass the limitations of the search function.

In our search, we focused on individuals and excluded companies such as media companies, record labels, and content farms, which are companies that produce large volumes of low-quality content (Bakker 2012; Maxwell 2011). In addition, we excluded content creators who are supported by broadcasting fees. In Germany, for example, some content creators belong to Funk, a public service content network belonging to two broadcasters, ARD and ZDF. We also excluded content creators who were originally known through other media, such as television and print, and those who had not been active for several months or years. In summary, we sought content creators who had grown independently on the platforms and are considered to be platform-dependent entrepreneurs (Cutolo and Kenney 2021).

To obtain the most authentic answers possible, our selection also depended on the language fluency of the research team. Therefore, we started with German-speaking content creators in Germany, Austria, and Switzerland and continued our search with English-speaking content creators in other parts of Europe, the United States of America, and Australia.

In our search, we emailed about 360 potential interviewees. The e-mail addresses were available in the YouTube profiles or on personal websites. Of those contacted, 60 responded. Of these, 22 cancelled due to time constraints or because they were not interested. Of the 38 remaining, we conducted an interview with 26. The interview participants were from Australia, Germany, Japan, Spain, Sweden, Switzerland, the United Kingdom, and the United States of America. Their thematic orientations were business, technology, sports, travel, aviation, languages, films and TV series, and education. (in Online) Appendices B and C provide an overview of the interviewees and their characteristics.

### Interview Process

The first cycle of interviews was conducted as in-depth, semi-structured interviews via Zoom between May and November 2021 and lasted an average of 60 min. We applied an interview guide and mirroring techniques (Myers and Newman 2007). All interviews were recorded and transcribed. A detailed description of the interview process, based on Myers and Newman’s (2007) guidelines, and the interview guide can be found in (in Online) Appendices D and E. After an initial assessment of the analysis and the preliminary results in April 2022, we started a second interview cycle from April to July 2022. We used a revised interview guide in a total of five more interviews that lasted an average of 45 min. These five interviews represent our theoretical sample and are marked with asterisks in (in Online) Appendix B.

### Data Analysis

To address our research questions, we used grounded theory techniques following Charmaz (2014) and applied constant comparative methods (Glaser and Strauss 1967), resulting in an iterative approach. First, we coded three interview transcripts line by line. We labelled text passages with short captions representing initial codes. An example would be’expanding channel focus’. We selected interviews with content creators who were as different from each other as possible to generate the maximum number of initial codes at an early stage. In addition to line-by-line coding, we used gerunds to describe the actions of the content creators. Charmaz (2014) and Glaser (1978) are proponents of coding with gerunds because they promote the coding of processes and actions instead of focusing on topics or individuals. Charmaz (2014, p. 245) describes that gerunds render common sequences and connections both within and between transcripts easier to recognise and ‘foster theoretical sensitivity’. Line-by-line coding resulted in many initial codes, which is why we applied focused coding after each of the three interviews and compared the initial codes with each other to arrange and highlight those codes that made the ‘most analytical sense’ (Charmaz 2014, p. 138) to our research problem. At the same time, we transformed descriptive and long initial codes into short, analytical ones (Charmaz 2014). Some emerging concepts were multilayer, which is why we also divided the focused codes into first- and second-order codes (Gioia et al. 2013). Examples include ‘figuring out how to grow’ as a first-order focused code and ‘becoming more professional’ as a second-order focused code. We analysed the other transcripts mainly with the focused codes because focused coding allows us to ‘examine large batches of data’ (Charmaz 2014, p. 138). If the focused codes did not cover text passages, we added new initial codes. In summary, we cycled through an iterative loop of initial and focused coding.

Throughout the data collection and analysis, thoughts, reflections, and possible theoretical starting points were recorded in memos. For example, after each interview, we reflected in a memo on the participants’ mood and location. At the beginning of the interview, one participant rushed through the room with the laptop and seemed hasty until sitting down. With another, technical malfunctions occurred, which is why the participants had to log in to a new meeting after 30 min. In the analysis, we also used memos to record thoughts and determine possible theoretical directions. We started writing memos with the first interview because it helped us develop the initial codes into more abstract focused codes. Integrating passages from the transcripts into the memos also helped us to recognise and counteract any preconceptions (Charmaz 2014). To further prevent preconceptions, we discussed the interviews within the research team and reviewed each other’s codes in a double-coding step. We used MAXQDA software for coding and memo writing. To increase the generalisability of our theory, what Urquhart et al. (2010, p. 372) refer to as ‘scaling up the theory’, we used a combination of clustering (Charmaz 2014), theoretical coding (Charmaz 2014; Urquhart 2022), theoretical sorting (Charmaz 2014), and diagramming (Charmaz 2014; Urquhart 2022) to organise the codes and memos visually and tested various possible relationships between the codes. With this approach, we identified similarities and consolidated emerging concepts into similar categories (Charmaz 2014). This was done on paper and visual collaboration platforms such as Miro.

We applied two types of theoretical sampling according to grounded theory and theory building. Theoretical sampling allowed us to expand the scope of emerging concepts and refine the properties and limitations of these concepts from the initial analysis with new data (Charmaz 2014). The first type, in classical understanding, is theoretical sampling, which took place later and aimed to verify the main categories (Charmaz 2014). For this purpose, we recruited five interview partners (CC05, CC06, CC07, CC18, CC23). The interviewees from the theoretical sampling were similar in personas but their videos dealt with thematic orientations in business and technology (Urquhart et al. 2010). The second type, referred to by Urquhart (2022, p. 159) as a ‘light form of theoretical sampling’, occurred throughout the data collection and arose from the answers and findings from previous interviews explored with later interviewees. Both forms helped us explore the characteristics, boundaries, and relationships of our preliminary categories (Charmaz 2014). We completed our data collection and reached theoretical saturation when no new properties and only more instances of the core categories appeared (Charmaz 2014; Urquhart 2022).

## Findings

To represent a chain of evidence, we have based our presentations on Gioia et al.’s (2013) data structure and combined it with the Charmazian terminology by denoting initial and focused codes (Charmaz 2014). The abundance of some findings allowed us to divide the focused codes into first and second orders (Gioia et al. 2013). The data structures are shown in Tables 3, 4, 5, 6, 7 and 8.

**Table 3:**
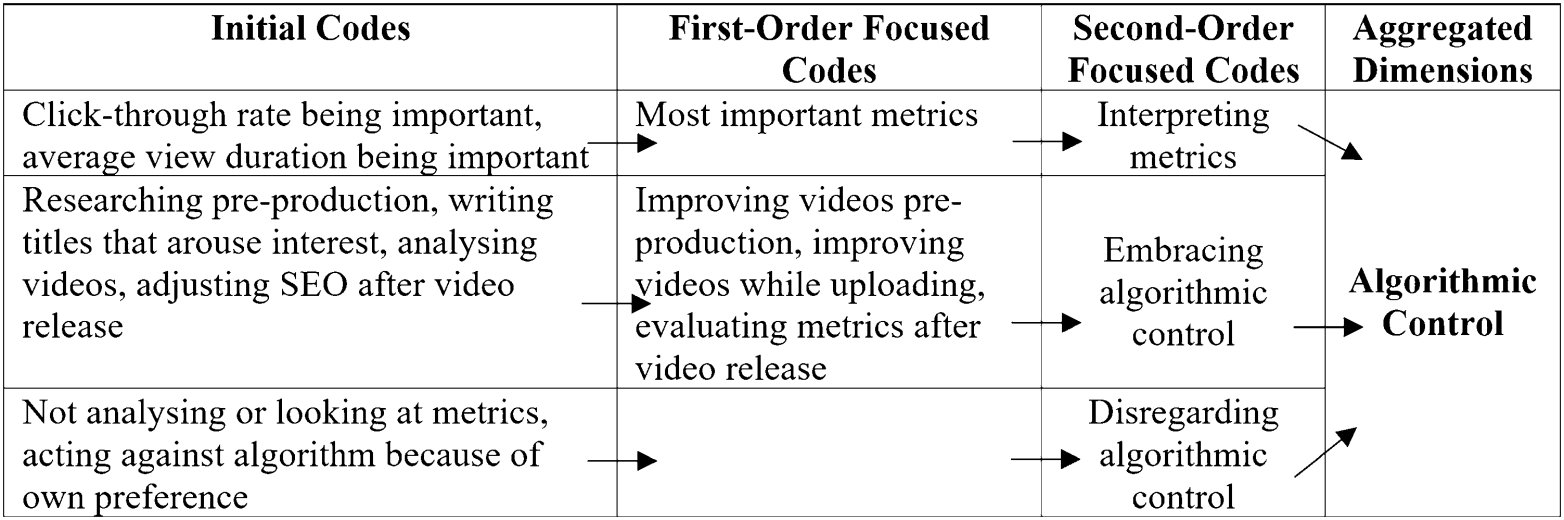
Data structure of algorithmic control

**Table 4:**
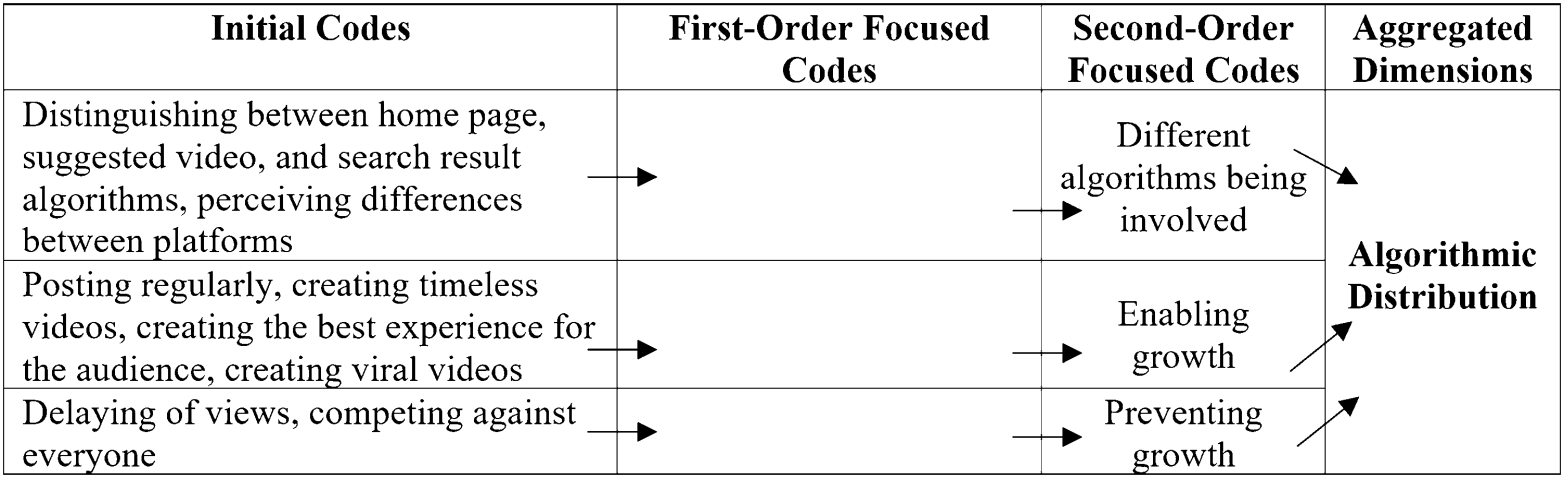
Data structure of algorithmic distribution

**Table 5:**
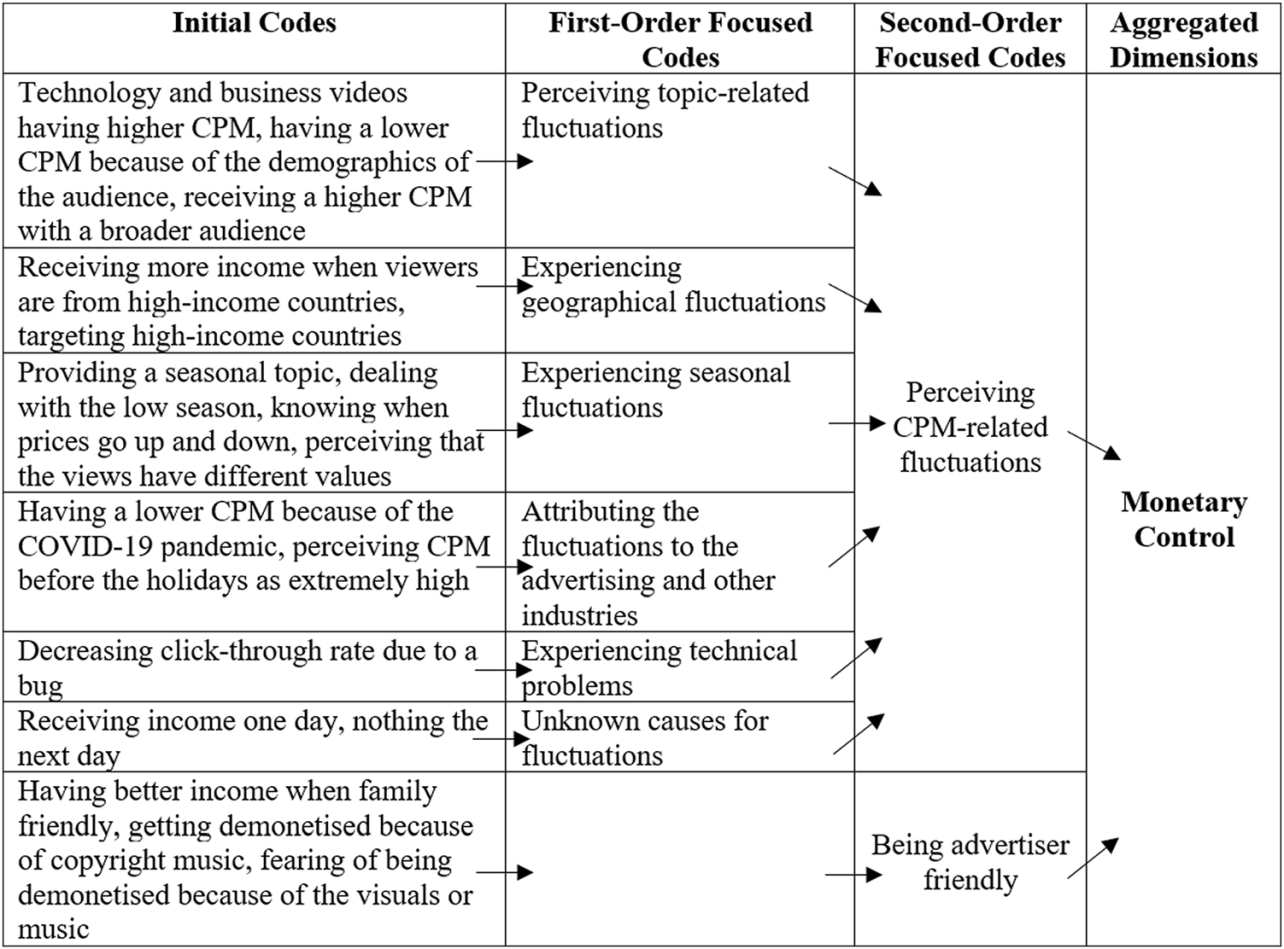
Data structure of monetary control

**Table 6:**
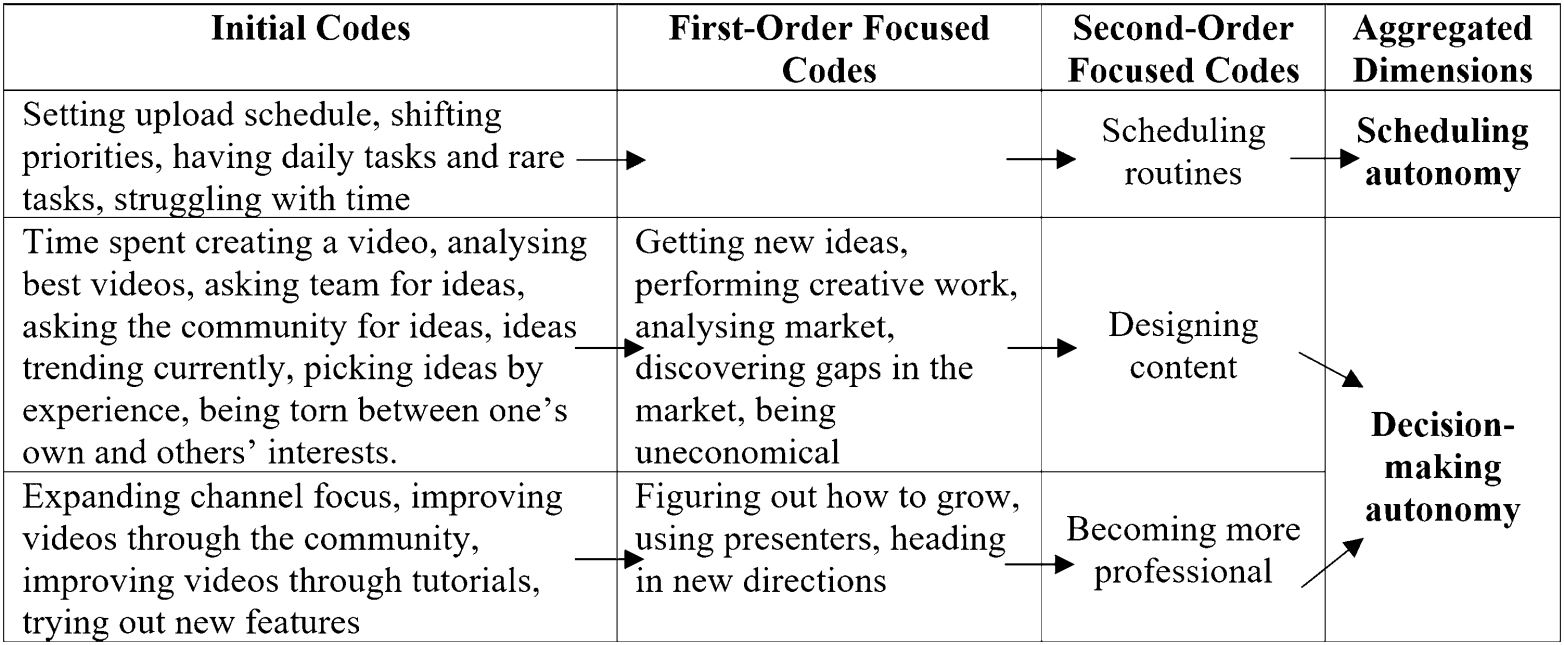
Data structure of perceived autonomy

**Table 7:**
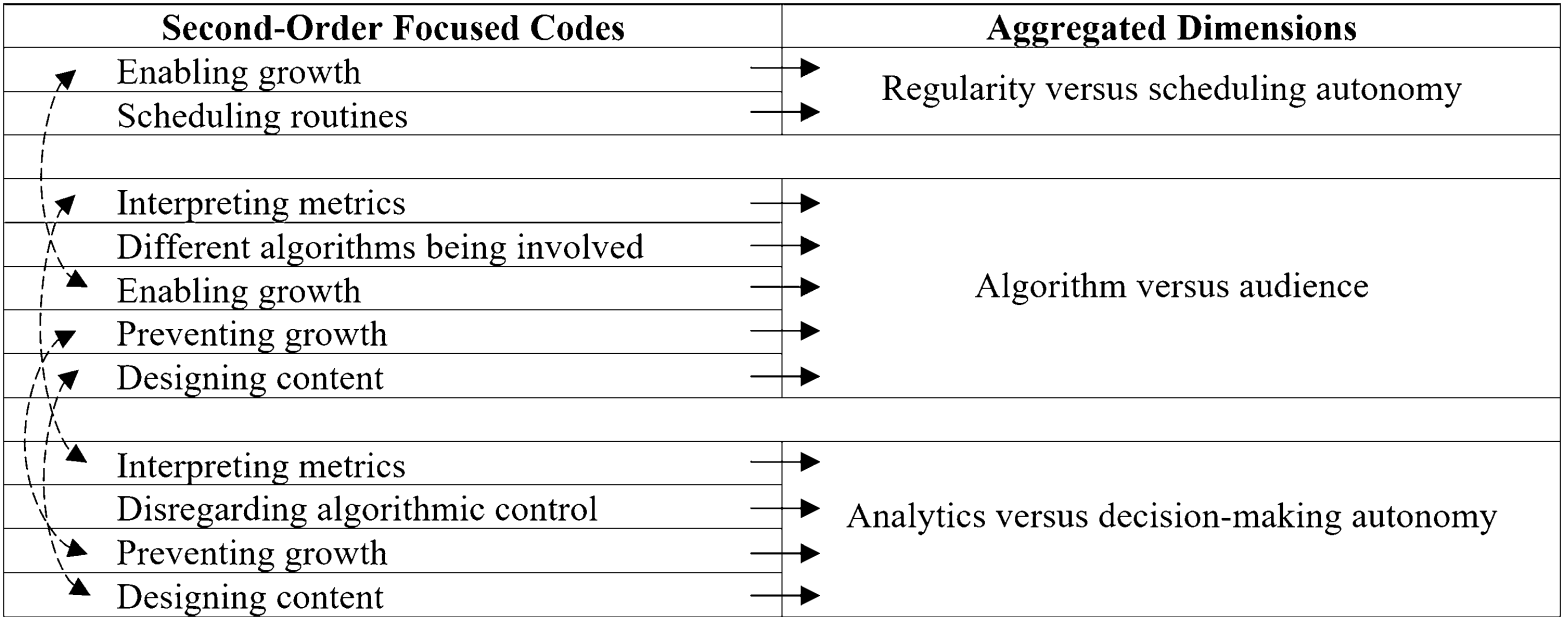
Data structure of tensions

**Table 8:**
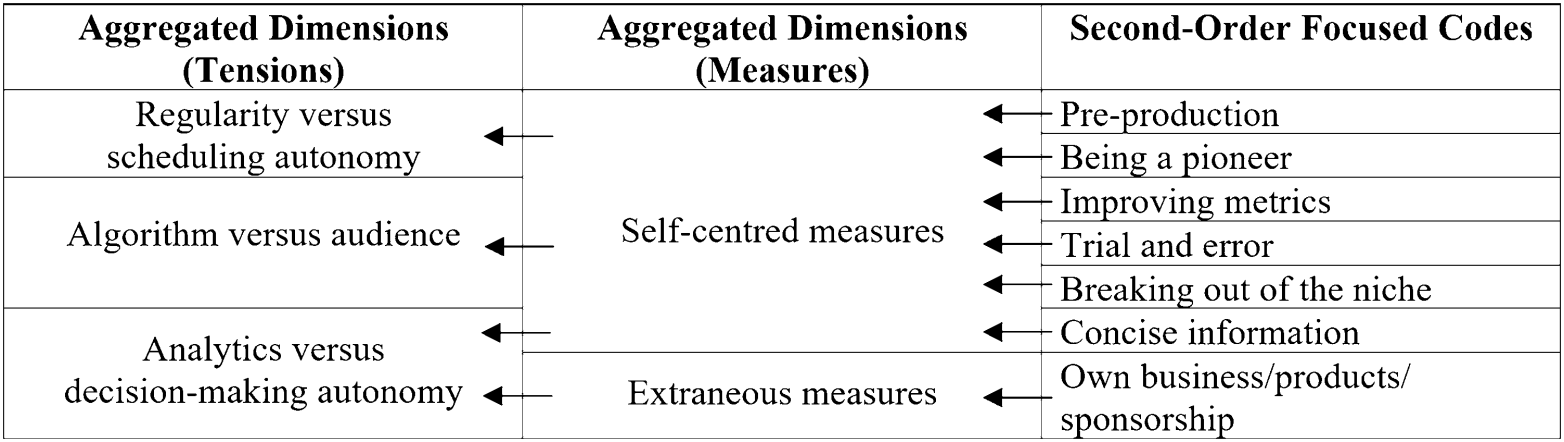
Data structure of measures

### Findings Part 1: Perceived Control

#### Algorithmic Control

In Table 3, algorithmic control is divided into three second-order focused codes: interpreting metrics, embracing algorithmic control, and disregarding algorithmic control.

##### Interpreting Metrics

In the interviews, the content creators^1^ repeatedly mention two metrics: the click-through rate and the average view duration, which they also refer to as watch time. Both metrics are considered important.

*It’s mainly two metrics. So, the first metric is going to be the click-through rate, which is like the most important. So that’s the amount of people that [sic] is going to click on your video when it’s like shown on YouTube basically. So, this you can influence by choosing a really good thumbnail or a really good title. . . . And then the second thing is the watch time, which is how long someone is going to watch the video. And you can influence this by making a video people are interested in* (CC23, Stayer).

At the same time, they felt they did not understand all the relevant key figures of the algorithm.

*I don’t think anybody really understands fully how the algorithm works. The way I see it is it’s like a learning, breathing machine. It’s like always changing to benefit both creators and YouTube as well, because mutual benefit by creators putting out content that’s valuable and that keeps people engaged for a long time. … I think it’s a very complex thing and no one really knows exactly how it works. But I think it boils down to just mostly watch time, and watch time is just how valuable your content is. People won’t watch a nonvaluable video all the way through* (CC06, Amateur).

##### Embracing Algorithmic Control

Content creators do not perceive the role of the algorithm necessarily as negative. Many content creators appreciated and embraced algorithmic control to improve their content. They used the analytics provided by the platform, for example, to improve keywords, titles, and thumbnails. To obtain suggestions for titles, they used external analysis tools. Some dealt in depth with the key figures and used A/B testing to find out which thumbnails were the most successful in improving the click-through rate over a few weeks. They described how they also looked closely at the videos to find out why viewers were leaving their videos to improve average view duration. This analysis was initially self-centred. For example, they tried to find out whether the sound quality was impaired or whether they were using words that were not familiar to the viewers before looking for factors outside of their control.

##### Disregarding Algorithmic Control

Overall, there was a wide range between those content creators who embrace algorithmic control and those who forgo it. Some told us that they looked at the metrics but did not aim for an in-depth analysis of the numbers and regularly checked just a few metrics. There are also statements suggesting that the role of algorithmic control could depend on the number of subscribers. As content creators built a community, the optimisation of metrics lost more and more importance, and content creators could now fall back on their experience:

*Now it’s pure experience. Experience also shows that optimisation tends to be a bit overrated if you have a certain size [number of subscribers]. If you don’t have it, it must be super well maintained. If you have a certain size, you will be recommended, and thus you will already outperform other YouTubers anyway* (CC10, Aspiring Professional).

##### Algorithmic Distribution

An important aspect of algorithmic control is the distribution of content. Table 4 provides an overview of the data structure.

*Different algorithms being involved*: Many content creators were on several platforms. They felt that the different platforms had different preferences and therefore that the algorithms were designed differently. One content creator said that TikTok, a short video platform, had an interesting algorithm because *‘really absolutely anyone can go viral’* (CC10, Aspiring Professional), meaning that a video is very popular. On YouTube, some content creators saw themselves as confronted with one algorithm, while others spoke of multiple algorithms: home page, suggested videos, and search results. Some reported that they focused on the search algorithm because it mainly used keywords and was the easiest to control. They also liked to use external tools to make their videos *‘perfectly SEO relevant’* (CC01, Amateur).

*Enabling growth*: Many of the content creators interviewed mentioned that regularity was rewarded. One person told us about their experience on Instagram and that they had noticed a sudden surge in followers when they were more active and uploaded a post daily. Another content creator agreed with this sentiment on YouTube. When they decided to do content creation full time, the numbers of views and subscribers increased every month. When content was regularly uploaded to the platform, they noticed that each of their videos received more traffic, which they attributed to the distribution of the algorithm. The regularity was mainly mentioned with Twitch and YouTube. A content creator said of Twitch:

*Twitch also rewards regularity, but above all [if] you have a lot of viewers, then you are at the top.… To all people who start with Twitch, I always give one piece of advice: Start with a different platform. On Twitch, it’s incredibly difficult to impossible to climb from natural growth alone* (CC10, Aspiring Professional).

*Preventing growth*: Content creators also noted that it was important to plan content for the long term because videos could still be picked up after years and offered to the target group: *‘We have videos that are a year old, and the curve was really flat, and now they go up all at once’* (CC01, Amateur).

Content creators could not explain this phenomenon and suspected a threshold had been exceeded, which was why the algorithm captured the video and distributed it on the platform. Furthermore, they reported that they had experienced a decline in the number of views if the algorithm could not assess their content. Examples included offering a new range of content to expand the channel variety and changing the channel name. When expanding channel variety, the videos received consistently lower views and only reached more users after two years. After changing the channel name, some content creators reported, the channel was reset and classified as a new channel. Although the content and style of the channel had not changed, the number of views and the growth of subscribers halved. Several content creators emphasised that competition was also crucial, and the algorithm favoured the best overall quality video. For content creators, it was increasingly difficult to stand out because the competition increased, but the quality of the content also improved.

#### Monetary Control

Two types of monetary control were detected: Cost per mille (CPM)-related fluctuations and advertiser-friendliness. CPM-related fluctuations are additionally ascribed to topics, geography, seasons, advertising and other industries, technical problems, and unknown causes. For all first-order focused codes, we list short examples. Table 5 provides an overview of the findings on monetary control.

##### Perceiving CPM-Related Fluctuations

The topic-related fluctuations affected both whether content creators addressed their target group appropriately with the videos and whether other channels uploaded videos on the same topic at the same time and whether videos competed with each other. Other interviewees reported that their CPM increased once they broadened their channel focus and their content was palatable to a larger audience. When content creators operated multiple channels, they also noticed differences. Content creators reported that compensation would be higher in technology-driven and business channels. However, if the target group is a demographic with little to no income, such as children, the CPM was also lower. A content creator with a broader reach told us that their greatest variance was geographical and that they received less for a video that was most viewed in India than for the same number of views in the United States of America.

*Like, once you have the audience that you’re targeting, the CPM doesn’t vary too much. Like, it’ll vary, but the biggest variances come with geography. … And so, if you want to be able to make a living as a social media creator, you know, it helps to target countries that have higher CPMs* (CC25, Expert).

Seasonal fluctuations are calendrical and relate to the topic. For example, explanatory videos receive more views before exams than during exam-free periods. The same applies to hearty cooking recipes on cold days compared to warm days. The fluctuations from advertising and other industries are related to the temporal marketing investments of companies and may occur seasonally and during crises. Many interviewees told us that during the holiday season in December, their CPM doubled compared to January. In the spring of 2020, when many European countries had imposed lockdown regulations due to the COVID-19 pandemic, their CPM fell massively. A content creator told us that the most critical games in the gaming industry are released between October and December, which is why they earn the most income at this time. Occasionally, technical problems occur. A content creator told us that the click-through rate dropped by 30 per cent for two days. A bug caused this, and the platform later fixed it. There are also fluctuations with unknown causes. One content creator’s successful video suddenly stopped generating revenue.

*When I did this [software] tutorial, I had 75 francs in one day. It’s great, of course. I mean, I also had courses cancelled, and money was lost. And of course, I was very happy, but that dropped to zero from one day to the next. At first, we really thought it was some mistake, a problem, and then we asked [YouTube]. And no, it was just like that. And that’s why I don’t want to build my business on it [YouTube]* (CC07, Amateur)*.*

Besides the fluctuations during the COVID-19 pandemic, technical problems, and unknown causes, CPM-related fluctuations repeated seasonally. Many content creators were familiar with the fluctuations of their channels and thematic orientation and hedged against them by taking precautions for bad times. Some also had contrary experiences. Many content creators who had a small reach had told us they had not been affected by income fluctuations. The number of views correlated with the payout. If they had fewer views, then they also had less revenue. Fluctuations in income only occurred when the content creators had not posted any content, and they could explain these slumps with their inactivity.

##### Being Advertiser Friendly

Many content creators also told us that they paid attention to not being classified as advertiser unfriendly or infringing copyrights. One described how YouTube regularly informed them about changes on the platform and would also demonetise or delete critical content that violated their policies. Content creators were also asked to complete a questionnaire before each upload indicating whether graphic violence, sexual content, or the like occurred in the video. These guidelines may cause doubts about whether certain content is appropriate. One content creator wanted to make a video about a book that showed a woman on the cover who was slightly exposed but still covered. Despite the harmless depiction, they had considered whether they had to redact the book cover and might hide certain representations in the book or not show them at all:

*And I asked to myself, then you’re going to make a black bar in front of it, right? … You sometimes get a bit paranoid when you’re in … a grey area of copyright. Is that still okay now? Should I make the cut now? … Because with YouTube, it can be over relatively quickly* (CC09, Aspiring Professional).

It is also reported that measures could be taken retroactively through the platform, and content was demonetised even though it had been on the platform for several months or years. These retroactive measures unsettled content creators because they could never be sure that the platform would not eliminate their income.

### Findings Part 2: Perceived Autonomy

Table 6 summarises the findings of perceived autonomy, which in turn is divided into scheduling autonomy and decision-making autonomy.

#### Scheduling Autonomy

Scheduling autonomy for content creators includes upload frequency, working hours, and scheduling tasks and workdays. Many of the interviewees described to us that they roughly plan when to upload content. They preferred certain days of the week or frequencies. However, all interviewees said they take it as a rule of thumb and could also deviate from them. Some also told us that they uploaded the content with a time delay to reach their audience in the best possible way. This can be in the evening or at weekends when the target group and community are active. Others uploaded their videos immediately after completion. This was preferable for tutorials found by most of the audience with the search function. For example, employees search for tutorials about software features during working hours. Unless the function or the software changes, the popularity of the videos lasts. All content creators described similar routines to us and aimed for a frequency that repeated daily, weekly, or monthly. Content creators who ran their own businesses in particular often bundled certain tasks and used certain times to produce multiple videos. They called this *‘batch recording’* or *‘batch editing’* (CC05, Amateur).

#### Decision-Making Autonomy

##### Designing Content

Decision-making autonomy includes how content creators design their content, what they show or discuss in their videos, and which audience they want to address. The content creators designed the content of their videos in different ways. Some considered wishes from the community, preferring ideas that would add value to most of their viewers. If topics might not suit all viewers, these content creators made the content available on second channels or different platforms. In some cases, there were tensions between their own content preferences and their audience’s. Others said their creative process involves other people from their team or environment. For example, one content creator meets weekly with their video editor to discuss ideas, projects, and tasks. Yet another employed a person who develops new video ideas strategically.

##### Becoming More Professional

Content creators appealed to different audiences. Either they could build a community, which happened linearly and steadily, or they could try to target a foreign audience with a specific algorithm (i.e. home page, suggested videos, and search results). Content creators with a smaller reach such as personas amateur and aspiring professionals described that their content often targeted the search algorithm and used keywords to optimise their videos to be found by users. Once a certain reach had been built, the search algorithm played a less important role, and the content creators could rely on the number of views by the subscribers. Another content creator told us that they were targeting the suggested video algorithm because these videos had greater potential to reach a large audience and *‘go viral’* (CC23, Stayer).

*Now when we create videos. I’m not doing videos for my subscribers. I’m making videos for people that don’t know me. …. Like I always say the same thing because I know that people … don’t know me, so I cannot assume. So, if I want the video to do well, I need to assume that the people that are going to watch it don’t know me and make it interesting for them* (CC23, Stayer)*.*

Decision-making autonomy also means that content creators decide who is seen in their videos. A content creator described that they had reduced their workload and introduced new presenters on their channel. However, this step had to be planned and done slowly so the audience could become accustomed to it. Viewers would not adapt to new people quickly, which is why they were still shooting videos but not as often as before. Over time, they could continue to withdraw because the new presenters had established themselves and even built up fanbases.

*My sister started shooting videos with me. We then uploaded videos of the two of us, and now two years later, people are also looking forward to the videos with my sister. … This doesn’t happen overnight. You just have to be careful with such changes* (CC21, Stayer).

#### Work Methods Autonomy

Work methods autonomy describes the format in which content creators are allowed to create their contributions. This includes camera, sound, and image resolution. Because none of the content creators described such specifications and the instructions for use on the platform’s website (YouTube Help: Upload videos. https://support.google.com/youtube/answer/57407?hl=en&ref_topic=9257439, Accessed 13 May 2022) confirmed this, work methods autonomy is evidently irrelevant to our research focus.

### Findings Part 3: Tensions

Tensions can arise between control by the platform and the autonomy of the content creators. These tensions are divided into three areas: regularity versus scheduling autonomy, algorithm versus audience, and analytics versus decision-making autonomy. Table 7 provides an overview of which second-order focused codes are associated with the causes of tensions. Two-sided arrows indicate multiple associations of focused codes with areas of tension.

#### Regularity Versus Scheduling Autonomy

According to the content creators, the algorithm rewards regularity, which was why many felt obliged to upload content regularly. To maintain regularity, content creators might sometimes need to change their schedule, and they might deviate from their regular working hours to produce new video content so that they pick up on time-sensitive topics and follow trends:

*My first vacation,… as a full-time content creator, was absolute hell. I thought it would be a great idea to set up a laptop and record videos somewhere … in the countryside where there is no internet. It was bad for everyone involved. My friends were angry that I just wanted to produce some videos all the time. But a new game came out. …. Luckily for me, this game flopped* (CC10, Aspiring Professional).

Whether this intervention in leisure time was perceived as disturbing by the content creators could not be determined because some also perceived it as part of their personality. Even if the constant presence and accessibility are only perceived as disturbing by a few, this feeling should still be classified as tension.

#### Algorithm Versus Audience

The content creators reported difficulties assessing whether their content was not supported by the algorithm or whether viewers were less interested in it:

*Obviously, it can be frustrating sometimes when it’s like: ‘Uff, videos are not doing well’. But, you know, that’s either a sign that YouTube needs to find the right audience, or it’s a sign that, you know, your audience just isn’t interested in that video* (CC25, Expert).

This uncertainty, which we see as tension, arose because the content creators perceived it as an interplay between algorithm and audience that ultimately affected the success of the video and resembled a pendulum. The algorithm distributes content to some of the users and sets the pendulum in motion. Then, these users react to the content, thus swinging the pendulum back. The power of the momentum with which it returns is determined by the intensity of the users’ reaction. If they interact intensively with the content with likes, comments, and playback time, the algorithm suggests the content to other users, and this may be repeated indefinitely until the interaction of the users with the video diminishes and the algorithm also stops its activity, which brings the pendulum to a standstill.

#### Analytics Versus Decision-Making Autonomy

Although content creators were able to see through the analytics which content was positively received by the users and had gained a large reach, some decided to disregard these metrics. They reported that they were consciously uploading videos that did not please the algorithm and could harm them:

*Most of the time, it’s clear to me in advance [why a video did not perform well], and I just bring it anyway because I’m up for it. But it doesn’t make sense for the algorithm. That’s a bit of stubbornness* (CC10, Aspiring Professional).

Some content creators felt limited in their room for manoeuvre because they could not freely express themselves in their content. They refrained from taking up serious topics and did not consider them because sophisticated content did not appeal to the audience enough:

*For example, I did a few topics where it was also about environmental protection. … Such a video is just not so well received because it is not such an entertaining one. … If I were now in the mood for [such] a video. … Then I don’t even bother to make the video because I know it won’t do so well, probably. And then I don’t even consider it* (CC16, Enthusiast).

### Findings Part 4: Measures

To manage the tensions described in the preceding section, content creators use a variety of strategies. These strategies are not applied consistently and have varying degrees of effectiveness. For content creators, these are attempts to demystify the behaviour of the algorithm and the audience. The various measures can be directed inwards or outwards, which is why we distinguish between self-centred and extraneous measures. Self-centred measures enable content creators to directly influence and control their behaviour. In contrast, extraneous measures arise through external support. Table 8 provides an overview of the various measures.

#### Regularity Versus Scheduling Autonomy

##### Pre-Production

Many content creators reported their experience of leisure or holidays and that they uploaded content despite their absence so that the algorithm did not ‘*forget’* them (CC19, Worker). This content was less up to date, but it was important to the content creators that the algorithm would notice that they were still active. A content creator compared YouTube to streaming platforms such as Twitch and added that streamers experience a disadvantage due to absence. In turn, content creators on YouTube could pre-produce videos and therefore had fewer consequences:

*Pre-production is a YouTuber’s most powerful tool. As a streamer, you really have a huge disadvantage because you are simply offline. … On YouTube, … you can offer content that is then less relevant–relevant in terms of time. … Unfortunately, this is possible, but the algorithm is fed* (CC10, Aspiring Professional).

Pre-production can contribute to scheduling autonomy, thus reducing the tension between regularity and scheduling autonomy. Pre-production seeks to satisfy both demands at the same time, which is equivalent to a both-and response strategy to manage tensions.

##### Being a Pioneer

Our research also observed that content creators strive to be pioneers and try out new features and platforms. One of the most striking examples was that content creators had interrupted their holidays to produce up-to-the-minute content or that they worked through the night to enable timely content creation. These actions had less impact on the upload frequency than on the content of the next upload, for which something else was originally planned. In the short term, other content was produced to maintain upload frequency and deliver the first video on this topic.

#### Algorithm Versus Audience

##### Improving Metrics

Many content creators described how they adjusted and adapted such elements as titles, keywords, and thumbnails after the content was online. This optimisation targeted the algorithm by connecting more suitable titles, keywords, and descriptions with the content. As a result, the optimisation made it easier for the algorithm to assign content to the right target group. Subsequently, the optimisation would also affect the audience if more appealing titles and thumbnails encouraged more users to watch the video.

*If your videos [are] not doing as well as you’d hoped or you’d expected, you can play around, change the thumbnail up, change the title, and because you’ve got kind of live analytics, you can see: ‘Okay, it’s actually doing better now’* (CC04, Amateur).

Although these measures are often taken, they do not completely resolve the tension between the algorithm and the audience, which means the algorithm remains a black box for content creators.

##### Trial and Error

Some content creators described how experimenting with the content is crucial. If they could not determine what led to a poorer reception of the content, they would try out new ideas to keep their audience interested in their content.

*I always try to experiment as much as possible because I always feel like, if you just keep making the same content over and over again, people are going to get bored. You never know what could resonate with a particular audience* (CC25, Expert).

This measure is mainly aimed at the audience to help ensure that the viewers remain interested and their engagement with the content is maintained. It also affects the algorithm, which in turn has to classify the new idea. Even with new content, this tension will remain or develop into a new field of tension: analytics versus decision-making autonomy. Content creators reported that new content received fewer views than their other content and was detected by the algorithm only with a time delay. Accordingly, the timing of a change is decisive, as is whether a change happens suddenly or permanently. For content creators, a balancing act remains between change and stability.

##### Breaking Out of the Niche

Many content creators emphasised the importance of targeted content. They reiterated that finding the right target group for their content was crucial. Some also reported that they had tried a lot in search of the right target group and, for example, also changed the language. They switched to English, which a much larger audience could understand and thus boosted the reception of their content. Others expanded their reach by broadening their existing content into new thematic areas. Both groups initially started in a niche and broke out of it with linguistic or thematic variation. The algorithm needs time to classify the content in such cases too, which in the worst case entails a long wait until the classification is done flawlessly.

#### Analytics Versus Decision-Making Autonomy

##### Concise Information

Platforms’ framework conditions and the key figures that are available to content creators provide incentives that influence behaviour. One such incentive influences the length of a video. At the time of the interviews, YouTube allowed a second advertisement to be placed in videos after 8 min. This might lead to longer videos being uploaded in general because a single video can generate higher advertising revenues. Some content creators preferred concise information in their videos and would discard unnecessary content during editing to keep their viewers engaged with the content for as long as possible. They reported that their viewers would better receive more concise information and that their metrics also supported this perception.

*The longer you can have someone engaged in watching your video, … is going to help your video do well [sic]. So, some much shorter videos, where I’m just giving kind of concise information quite quickly, have done well.* (CC04, Amateur)

This tension manifests itself in three areas: analytics, incentives, and decision-making autonomy. Although the analytics support decision-making autonomy, in this case, there can be tension with the incentive for longer videos that is not completely resolved by the content creator.

##### Own Business/Products/Sponsorship

It was striking that many content creators had various income streams, and many ran their own businesses or sold their own products. Two of the content creators interviewed had already dealt with the question of what would happen to their channels if they had an accident or could no longer shoot videos due to illness. Because they did not want to be dependent on platforms or sponsorship, they founded a company on the side which operates separately from their channels. Others also emphasised that it is very important to look for reliable partners outside of YouTube in order to establish long-term collaborations and derive income streams from several sources.

## Model and Theoretical Integration

**Fig. 1:**
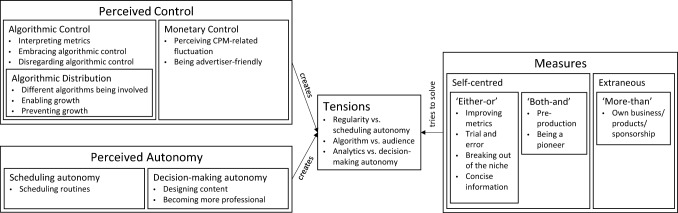
Field of tension in content creation on revenue-sharing social media platforms

The model presented in Fig. 1 shows how the four aggregated dimensions of perceived control, perceived autonomy, tensions, and measures are related to each other. Perceived control is located on the upper left side and includes algorithmic control and monetary control. Within algorithmic control is algorithmic distribution, which is a direct effect of algorithmic control. On the lower left is the perceived autonomy with scheduling and decision making. At the centre of Fig. 1 are the tensions arising from perceived control and perceived autonomy. The right side of Fig. 1 presents the measures taken as a direct response to the tensions. The measures are divided into self-centred and extraneous. Additionally, they are assigned to either-or, both-and and more-than responses (Putnam et al. 2016).

Our research contributes to the complementarity of algorithmic control, including the new construct of algorithmic distribution, scheduling, and decision-making autonomy for social media platforms, the resulting tensions affecting content creators, and the concrete measures associated with these tensions. Our contribution is illustrated in Table 9. In addition to the fine-grained distinctions within our aggregated dimensions, algorithmic distribution expands previous understanding of algorithmic control (Möhlmann et al. 2021; Wiener et al. 2021). It also differs clearly from algorithmic matching on online labour platforms (Möhlmann et al. 2021) and information diffusion among users of social media platforms (Stieglitz and Dang-Xuan 2013) by being a direct effect of algorithmic control. Algorithmic distribution ties in with other studies of user perception and folk theories about curation algorithms (Alvarado et al. 2020; DeVito et al. 2017; Eslami et al. 2016), where users puzzle over the distribution of content. We also provide in-depth insights about monetary control and perceptions of CPM-related fluctuations that extend the existing concepts of dynamic pricing, which is dependent on time, place, and display (Asdemir et al. 2012; Choi et al. 2020; Möhlmann et al. 2021). Our findings show that dynamic pricing is multifaceted and consists of topic-related, geographical, seasonal, advertising and other industries, technical and unknown fluctuations. In addition, our findings on being advertiser friendly relate to the performance rewards and performance punishments on multisided platforms (Lin et al. 2022). Our findings about scheduling autonomy and decision-making autonomy tie in with existing literature and expand it with the perspective of content creators on social media platforms. Content creators feel the need to become more professional, design their content independently, and develop work routines and habits individually (Polites and Karahanna 2013). Our findings on tensions have similarities with tensions reported in existing studies on online labour platforms, in that algorithm-versus-audience tension is similar to the work compensation tension between uncertainty and repeatability, because content creators and Uber driver have difficulties understanding the logic of the algorithm (Möhlmann et al. 2021). Analytics-versus-decision-making-autonomy tension is similar to the work execution tension between autonomy and supervision (Möhlmann et al. 2021) because the preferences of analytics limit autonomy. Regularity-versus-scheduling-autonomy tension connects to the ‘techno-invasion of technostress’ (Adam et al. 2017, p. 280) and ‘digital detox’ (Mirbabaie et al. 2022, p. 239) because the activities regularly required by the platform (Arriagada and Ibáñez 2020) blur the boundaries between work and private life. Our depth of findings about self-centred and extraneous measures is unique. Möhlmann et al. (2021) show bypassing and switching on labour market platforms as market-like behaviour and striking and embracing as organisational-like behaviour. However, neither concept offers a direct response to our tensions and cannot be transferred. Other studies have found that content creators are ‘leveraging algorithms’ (Kellogg et al. 2020, p. 391) and ‘working around the algorithm’ (Curchod et al. 2020, p. 17) by constantly uploading (Arriagada and Ibáñez 2020), A/B testing (Cotter 2019), and diversifying their incomes (Cutolo and Kenney 2021).

**Table 9:** Theoretical integration of our findings in the information systems and broader literature

**Findings**	**Refers to factors from the information systems literature**	**Refers to factors outside the information systems literature**
**Algorithmic control**	Algorithmic control (Möhlmann et al. 2021), guiding algorithmic control (Wiener et al. 2021)	Invisible cage (Rahman 2021)
Interpreting metrics	Technostress (techno-overload) (Adam et al. 2017)	Algorithmic recording (Kellogg et al. 2020)
Embracing algorithmic control		Algoactivism (leveraging algorithms) (Kellogg et al. 2020), visibility game (Cotter 2019)
Disregarding algorithmic control		Algorithmic resistance (Velkova and Kaun 2021), Algoactivism (non-cooperation) (Kellogg et al. 2020)
**Algorithmic distribution**	Algorithmic matching (Möhlmann et al. 2021), information diffusion (Stieglitz and Dang-Xuan 2013)	Algorithmic curation (DeVito et al. 2017; Eslami et al. 2016), algorithmic imaginary (Bucher 2017), algorithmic media (Ytre-Arne and Moe 2021), algorithmic prioritisation (Vaccaro et al. 2018)
Different algorithms being involved		User perception of algorithmic recommendations (algorithmic awareness) (Alvarado et al. 2020)
Enabling growth	Learning, network effects (L. Qiu et al. 2015)	Algorithmic rewarding (Kellogg et al. 2020), uploading constantly (Arriagada and Ibáñez 2020)
Preventing growth		Winner-take-all (Cennamo and Santalo 2013; Gawer 2014), algorithmic rewarding (Kellogg et al. 2020)
**Monetary control**	Incentives, revenue sharing (Tang et al. 2012)	Power asymmetries at the governance level (Curchod et al. 2020, p. 14)
Perceiving CPM-related fluctuations	Dynamic pricing (Asdemir et al. 2012; Choi et al. 2020; Möhlmann et al. 2021), technostress (techno-uncertainty, techno-unreliability) (Adam et al. 2017; Mirbabaie et al. 2022)	Dynamic pricing (Kumar and Sethi 2009)
Being advertiser friendly	Performance rewards, performance punishments (Lin et al. 2022)	Advertiser-friendly, punishment (Caplan and Gillespie 2020)
**Scheduling autonomy**	Scheduling autonomy (Ye and Kankanhalli 2018)	Scheduling autonomy (Morgeson and Humphrey 2006)
Scheduling routines	IS habits (temporal context, physical context, social context, task definition, mood, other antecedent states) (Polites and Karahanna 2013)	Recurrent interaction patterns (Becker 2005)
**Decision-making autonomy**	Decision-making autonomy (Ye and Kankanhalli 2018)	Decision-making autonomy (Morgeson and Humphrey 2006)
Designing content		
Becoming more professional		
**Tensions**	Tensions (Mini and Widjaja 2019)	Tensions (Putnam et al. 2016)
Regularity vs scheduling autonomy tension	Technostress (techno-invasion) (Adam et al. 2017), digital detox (Mirbabaie et al. 2022)	Uploading constantly (Arriagada and Ibáñez 2020)
Algorithm vs audience tension	Work compensation tension (uncertainty vs repeatability) (Möhlmann et al. 2021)	
Analytics vs decision-making autonomy tension	Work execution tension (autonomy vs supervision) (Möhlmann et al. 2021)	Power asymmetries at the governance level (Curchod et al. 2020), power relations (Kopf 2020)
**Self-centred measures**		Working around the algorithm (Curchod et al. 2020)
Improving metrics		Algoactivism (leveraging algorithms) (Kellogg et al. 2020), A/B testing (Cotter 2019), algorithmic recording (Kellogg et al. 2020)
Trial and error		Algorithmic gossip (Bishop 2019), visibility game, A/B testing (Cotter 2019), algoactivism (leveraging algorithms) (Kellogg et al. 2020)
Breaking out of the niche		
Concise information		
Pre-production		Uploading constantly (Arriagada and Ibáñez 2020)
Being a pioneer		
**Extraneous measures**		Working around the algorithm (Curchod et al. 2020)
Own business/products/sponsorship		Income diversification (Cutolo and Kenney 2021)

Table 9 provides a comparison of our findings with the literature to date. We also differentiate between literature within and outside information systems. Literature outside information systems comes from such disciplines as management, computer science, and sociology.

## Theoretical Implications

This study answered three main research questions:

1. How do the algorithms of social media platforms influence content creators’ behaviour?

Our data show that content creators on social media platforms are a heterogeneous group. Accordingly, they respond to algorithmic control in different ways. Content creators with fewer subscribers, such as those we characterised as amateurs and aspiring professionals, use the general strategy of providing content that can be found through the search feature. This may give them a sense of control. Many people who want to expand their reach also rely on external tools and tests. Previous literature has already identified A/B testing (Cotter 2019), algorithmic gossip (Bishop 2019), and leveraging algorithms (Kellogg et al. 2020) as coping mechanisms. These tools are less important for established content creators such as those we characterised as stayers, skyrockets, and experts because they mostly produce content for their subscribers, who watch their content regularly. Nevertheless, established content creators are also exposed to fluctuations in the numbers of views and could not explain certain developments in the numbers to us. These fluctuations can be reduced by regular uploads, which are also pre-produced, and the amount of content (Arriagada and Ibáñez 2020). It also shows that content creators face a ‘winner-take-all’ situation (Cennamo and Santalo 2013, p. 1331; Gawer 2014, p. 1241). This is particularly pronounced on the platform Twitch because new and small content creators described difficulties in growing organically. This also explains why content creators with fewer subscribers use tools and do everything in their power to optimise their content. In contrast, more established content creators do without it or draw on their experience. In general, content creators can be divided into three groups depending on how they adjust their content: search algorithm-driven, recommendation algorithm-driven, and community-driven.

2. How does revenue sharing on social media platforms influence content creators’ behaviour?

Our research shows that content creators adapt their behaviour (cf. Cram et al. 2020; Möhlmann et al. 2021; Wiener et al. 2021) by either avoiding or bowdlerising certain content, approaches that can even be interpreted as self-censorship. Thus, they focus on content and topics that have delivered good key figures in the past, similar to algorithmic rewarding (Kellogg et al. 2020). Our data demonstrate that incentivisation does not primarily affect the quality of content but its supply (cf. Chen et al. 2019; Liu and Feng 2021). Thematically similar content is promoted, and diversity is limited. Some content creators have told us they have already considered whether their content violates the community guidelines and whether they need to adapt their content accordingly. For example, the platforms do not distinguish content that serves to educate people, such as news coverage, sex education, and war crimes, which means that this content is also negatively affected. In addition, the community guidelines of the social media platforms are also culturally characterised mainly by a US-centred or Americentric worldview, which may restrict content with contrasting perspectives and opinions from other cultures and countries. Many content creators also told us that they paid attention to being classified as ‘advertiser friendly’ (Caplan and Gillespie 2020, p. 4). Apart from the community guidelines, platforms do not provide rules (Cotter 2019) for content, which is why content creators remain relatively free in their content creation. However, incentivisation still certainly promotes or punishes content (Caplan and Gillespie 2020; Lin et al. 2022). Our findings show that the content quality increases through the content creators’ experience over time and is not promoted by incentivisation. Revenue sharing is perceived as unreliable in encouraging content creation, which is why content creators rely on other sources of income. Overall, the income from social media platforms accounts for a small part of the total income streams. In this respect, we confirm Tang et al. (2012)’s study that incentivisation is an additional incentive besides reputation and exposure. We also reinforce Lin et al.’s (2022) study that punishments continue to be effective and content creators deliberately try to be classified as advertiser friendly (Caplan and Gillespie 2020, p. 4).

- How do content creators adapt to algorithm adjustments of social media platforms to ensure a reliable income?

Because platforms and their control mechanisms represent a complex environment, content creators who earn their income on these platforms try to illuminate the complex control mechanisms with various measures to discover which strategies help them. They seek to shine a light inside the black box by trying out new content, improving metrics, and professionalising the presentation of their content. These measures do not always have to be effective, but they are coping mechanisms (cf. Putnam et al. 2016) for content creators to counteract the powerlessness caused by the paradoxical tensions of algorithmic control and incentivisation. When resolving tensions, content creators first resort to self-centred factors that they can influence themselves before considering extraneous elements.

3b. How do content creators adapt to revenue-sharing adjustments of social media platforms to ensure a reliable income?

We found that a surprisingly large proportion of content creators diversify their income (Cutolo and Kenney 2021) to avoid dependence on certain platforms. This is related to the fluctuations caused by the CPM. Although some fluctuations, such as those caused by seasonal changes and advertising and other industries, are repeated, the proximity to the advertiser unsettles the content creators. Moreover, the unpredictability of the platform, which some content creators have experienced, also deters them from further expanding their engagement on the platform. Both issues lead content creators to develop different revenue streams (Cutolo and Kenney 2021) and reduce their engagement with the platform.

In sum, our study explains the algorithms’ influence on the behaviour of content creators on revenue-sharing social media platforms. In Gregor’s (2006) landmark typology of theories, our study contributes a Type II theory. Our findings expand the platform literature by applying multilevel thinking and providing a cross-level theory of transaction platforms because we provide insights into how organisational factors influence the behaviour of individuals (Klein et al. 1994). Furthermore, our findings shed light on content creators’ perception of control and autonomy and enrich the tension literature with insights into social media platforms. Our study also offers further evidence on strategies for resolving tensions and empirically reinforces some of Cutolo and Kenney’s (2021) diversification strategies.

Content creators are largely independent of platforms and autonomous in content creation. Through their diversification strategy, by generating income from different sources, they can be seen as independent entrepreneurs (Cutolo and Kenney 2021). Nevertheless, their content is shaped and influenced by the audience, advertisers, and algorithms. Our research shows that content creators are sometimes more and sometimes less influenced by algorithms. These findings have implications for strands of literature dealing with platforms, control, extrinsic motivation, and tension.

## Practical Implications

Our study has practical implications for social media platform owners and content creators. An important issue for platform owners is whether they should provide a revenue-sharing mechanism to the content creators. Our study shows that revenue sharing has an impact on the behaviour of content creators but that it is not sufficient to earn a living. Instead, professional-minded content creators have to build a more complex income model that incorporates several sources (Cutolo and Kenney 2021). In addition, our study gives content creators hints on how to act on social media platforms to successfully reach many users and potentially turn them into followers. Because we interviewed many content creators of diverse impacts and topics, our results may be seen as describing some best practice for distributing content through platforms: improving click-through rate and average view duration, starting in a niche, and targeting countries with higher CPM. Our findings clearly show that beginning content creators such as aspiring professionals in, particular should carefully analyse and react to the algorithms. Established and successful content creators may rely more on their follower base and, of course, on their experience of what works and what does not.

## Limitations and Future Research

Our study contains four main limitations. First, our efforts portray as many different voices as possible to reflect the heterogeneity of the field (Myers and Newman 2007). Despite these efforts, we cannot guarantee complete diversity, especially because it was challenging to talk to established individuals whom we described as stayers, skyrockets, and experts. Our selection also depended on the language skills of the interviewers, which is why the majority of the content creators were from high-income economies such as Germany, Switzerland, and the United States of America. Second, platforms are constantly and rapidly changing, so our findings only cover the period from May 2021 to July 2022. Because YouTube and Twitch, the central platforms of our research, were founded in 2005 and 2011 and were operated by tech giants Google LLC/Alphabet Inc. and Amazon.com, Inc. at the time of the study, we estimate that our findings may reflect the situation across several years. There have also been repeated announcements that Instagram, which is currently owned by Meta Platforms, Inc., is also considering offering revenue sharing to its content creators (Barinka 2022). Third, the data collected is self-reported. As a result, only the views of the content creators are represented, which may be biased and thus not accurately reflect reality. For example, we may have been given answers that satisfy social desirability or maintain conformity. Fourth, any kind of qualitative research has room for interpretation. Although we have increased reliability and intersubjectivity through double-coding, we interpret the world that the subjects have previously described to us, which means the subjects interpret their world, and we then interpret their interpretation (Myers and Newman 2007). In addition, the interviews are rare events for subjects, which also seemed unusual due to the digital interface of Zoom. The COVID-19 pandemic cushioned this circumstance because video conferencing became the new normal during the pandemic. Moreover, our respondents are generally tech-savvy thanks to their work.

Our study is based exclusively on content creators’ self-reported data, which hinders generalisability and results in a one-sided view. Future research could focus on methodically evaluating our findings. Due to the heterogeneity of the field, future research could address thematic subareas, uniform groups, and countries that are not high-income economies.

## Conclusion

Our study explores how algorithms can influence content creators’ behaviour. We examined the effects algorithms have on the behaviour of content creators, the importance of incentives, and how content creators ensure a reliable income. We conducted interviews with content creators and found conflicts of interest in paradoxical tensions between control and autonomy. Our study demonstrates that algorithmic control and incentivisation create paradoxical tensions that affect the autonomy of content creators. These paradoxical tensions involve algorithm versus audience, regularity versus scheduling autonomy, and analytics versus decision-making autonomy. Content creators attempt to minimise these paradoxical tensions by self-centred and extraneous measures. Self-centred measures include improving metrics, pre-production, and being a pioneer. Extraneous measures include creating other businesses, selling products, and accepting sponsorships. We propose a model that shows how and why tensions occur in revenue-sharing social media platforms and what measures are applied. Our findings provide valuable insights into the actions and views of content creators and contribute to the existing literature on platforms, control, extrinsic motivation, and tension. Our efforts provide implications for the growing industry of platform work and indicate how society is affected by algorithms.

## Supplementary Information

Below is the link to the electronic supplementary material.

Supplementary file1 (PDF 931 kb)

## Footnotes

1 To provide gender-neutral representation, we refrain from gender-specific pronouns. If necessary, we use ‘they, them, their’ as a singular antecedent.
