# Supplementary material for: Content Creators Between Platform Control and User Autonomy: The Role of Algorithms and Revenue Sharing
Source: Bus Inf Syst Eng. 2023 May 24:1–23. Online ahead of print. doi: 10.1007/s12599-023-00808-9 (PMC10208186; doi:10.1007/s12599-023-00808-9)
Supplement: Supplementary file 1 — Supplementary file1 (PDF 931 kb) [file 12599_2023_808_MOESM1_ESM.pdf]

Content Creators Between Platform Control and User Autonomy –

The Role of Algorithms and Revenue Sharing

Tatjana Hödl, Thomas Myrach

Business & Information Systems Engineering (2023)

Appendix (available online via http://link.springer.com)

Appendix A: Personas of content creators

	Aspiring Professional	Stayer	Expert
**Start**	They started a YouTube channel during high school as a hobby for gaming, later more for entertainment and vlogging.	They originally started a YouTube channel for fun.	Their YouTube channel originally started as a hobby, and they were uploading videos under a pseudonym which they still use.
**Content**	Their videos are usually from one perspective and minimally edited.	In the beginning, their content was from various topics and similar to others. Then, their content became more focused on one area of expertise.	Their content was specific to one topic and became broader over the years.
**Growth**	Thanks to early and continuous content creation on YouTube, they were able to pursue careers in traditional media (radio, television) after high school.	They did not gain many subscribers with a single video but over a longer period.	Because of their large community and their inclusion in content creation, videos are watched steadily.
**Income**	The money earned from YouTube is the main component of their income.	Occasionally, their videos are sponsored. If so, it is by long-term partners.	Nowadays, YouTube is their only source of income besides merchandise and brand stores.
**Inspiration**	Aditotoro, Athina	muchelleb, Observe	PewDiePie, How To Cook That

	Amateur	Enthusiast	
**Start**	They started on YouTube a couple of years ago.	They started on Instagram and grew their account over a couple of years.	
**Content**	Their content is focused on one specific topic. They like to interact with their regular viewers in the comment section. They seek advice and inspiration in the community and engage in different groups on Facebook, Reddit, and Instagram.	They use YouTube because sometimes they want to talk about certain topics for longer than a few seconds/minutes. Their content was and still is specific to one topic. When they were interested in other topics, they opened a separate account.	
**Growth**	Their channel has a few subscribers, and most videos have only a couple of hundred views. Occasionally, their videos get a couple of thousand views.	After reaching 1 million followers on Instagram, they quit their day job. By doing so, they extended their portfolio and expanded their private YouTube account.	
**Income**	They receive little to no income from YouTube.	Their income comes mainly from brand deals and collaborations with brands.	
**Inspiration**	Baking With Elliott, Charlotte Kreft	Fashioninflux, Keltie O’Connor	

	Worker	Skyrocket	
**Start**	They have recently started on YouTube. They started a YouTube channel as a hobby to educate people on their craftsmanship.	They originally started a YouTube channel for fun.	
**Content**	They have a main occupation and mainly show people what they do and answer questions in their videos. Their videos are mainly voice-overs and edited minimally. They prefer to address questions in videos directly.	In the beginning, their content was from various topics and became more focused but still broad.	
**Growth**	Due to some videos that went viral and received several million views, they gained many subscribers on YouTube.	Due to a video that went viral, they gained a lot of subscribers on YouTube. Occasionally, their videos are sponsored. That is when they invest in editing. Otherwise, they keep it minimal.	
**Income**	The money earned from YouTube is an additional income used on special occasions.	Besides their studies, YouTube is their main occupation, and they were able to move out with their partner.	
**Inspiration**	S&S Horseshoeing, Patrick (H) Willems	ellbat, Beau Miles	

Appendix B: Overview of the interviewees

Pseudonym	Persona	Thematic orientation	Impact (subscriber count)	Involvement (upload frequency)	Estimated views per video	Duration (minutes)
CC01	Amateur	Adventure, sports	< 20,000	Weekly	10.000	69
CC02	Amateur	Entertainment	< 20,000	Biweekly	15.000	50
CC03	Amateur	Lifestyle, beauty, fashion	< 20,000	Weekly	15.000	60
CC04	Amateur	Photography	< 20,000	Biweekly	3.000	44
CC05*	Amateur	Business, technology	< 20,000	Weekly	400	58
CC06*	Amateur	Business, finance, real estate	< 20,000	Monthly	7.000	33
CC07*	Amateur	Business, technology	< 20,000	Weekly	800	65
CC08	Aspiring Professional	Adventure, sports	20,000 – 100,000	Weekly	40.000	67
CC09	Aspiring Professional	Films, TV series, Anime	20,000 – 100,000	Daily	15.000	85
CC10	Aspiring Professional	Gaming	20,000 – 100,000	Daily	20.000	75
CC11	Aspiring Professional	Politics, entertainment	20,000 – 100,000	Weekly	40.000	75
CC12	Aspiring Professional	Football training	20,000 – 100,000	Daily	8.000	52
CC13	Aspiring Professional	Travel	20,000 – 100,000	Weekly	100.000	12
CC14	Enthusiast	Cooking	100,000 – 500,000	Weekly	10.000	38
CC15	Enthusiast	Learning, school, motivation	100,000 - 500,000	Weekly	1.000	60
CC16	Enthusiast	Travel, culture	100,000 – 500,000	Weekly	80.000	65
CC17	Enthusiast	Aviation	100,000 – 500,000	Weekly	15.000	52
CC18*	Enthusiast	Business, technology	100,000 – 500,000	Biweekly	15.000	45
CC19	Worker	Music	100,000 – 500,000	Monthly	30.000	60
CC20	Worker	Books, publishing	100,000 – 500,000	Monthly	7.000	71
CC21	Stayer	Languages	500,000 – 1,000,000	2 to 3 times a week	8.000	63
CC22	Stayer	Films, TV series	500,000 – 1,000,000	Daily	15.000	42
CC23*	Stayer	Business, investments	500,000 – 1,000,000	Weekly	120.000	59
CC24	Skyrocket	Statistics, education	500,000 – 1,000,000	Monthly	30.000	87
CC25	Expert	Languages	> 1,000,000	2 to 3 times a week	500.000	58
CC26	Expert	Aviation	> 1,000,000	Weekly	800.000	76

** theoretical sampling*

Appendix C: Characteristics of the interviewees

Characteristics (n = 26)	Count	%	Characteristics (n = 26)	Count	%	Characteristics (n = 26)	Count	%
**Age (years)**			**Language of the videos**			**Country of residence**		
20-30	13	50	English	10	38	***Europe***	*19*	*73*
31-40	10	38	German	10	38	Germany	9	35
41-50	3	12	French	1	4	Switzerland	7	27
			Swiss German	1	4	Spain	1	4
			Others	4	15	Sweden	1	4
						United Kingdom	1	4
			**Language of the interviews**			***North America***	*5*	*19*
			English	11	42	United States of America	5	19
			German	11	42	***Others***	*2*	*8*
			Swiss German	4	15	Australia	1	4
						Japan	1	4

Appendix D: Interview Process according to Myers and Newman (2007)

Guideline 1: Situating the researcher
The team of interviewers consisted of three master's degree students and one doctoral student. Every member of the team of interviewers was enrolled or has completed the master's program in business administration. As a bachelor's degree, the members have different backgrounds (business administration, linguistics). The age ranged between 20 – 35 years, and three out of four members were female. All members were Caucasian and of Swiss nationality. The master's degree students conducted interviews as part of their master's thesis, which is normally due at the end of the study program, while the doctoral student acted as the project leader. Apart from conducting self-contained interviews, the doctoral student was also overseeing the interviews conducted by the master's degree students because most had little experience in the field.
**Guideline 2: Minimise social dissonance**
23 interviews were conducted online using videotelephony software programs, i.e., Zoom. The team of interviewers was casually dressed to mimic the interviewee's appearance. To make the interviewee comfortable, the members abstained from using virtual backgrounds and creating a false impression. Instead, neutral backgrounds like a white wall were seen. The language spoken during the interviews was chosen by the interviewee and was either (Swiss) German, or English. Because our knowledge of French was not sufficient to converse on complex topics, we held one interview in English by mutual agreement. Again, to make the interviewee comfortable, the members abstained from complex language and used similar language and jargon as the interviewee. The three remaining interviews were conducted either in person on-site, by phone, or via Instagram's messaging feature with text and voice messages.
**Guideline 3: Represent various 'voices'**
The procedure described in the chapter on data collection (i.e., determination of personas) for recruiting the interviewees allowed us to bypass the elite bias (Myers and Newman 2007, p. 22) and hear different perspectives on the same problem.

Guideline 4: Everyone is an interpreter
All interviews had been tape-recorded and transcribed in a timely manner. Additionally, notes were taken during the interviews and used during transcription for further clarification. After transcription, the team of interviewers read through each other’s interviews to avoid interpretations of the interviewees’ views and statements. If clarification was needed, the interviewee was contacted. Upon request, the transcript was sent to the interviewees for review. As described in the chapter on data analysis, we have prevented intersubjectivity and preconceptions through discussion within the research team and double coding.
**Guideline 5: Use of models in questions and answers**
The team of interviewers used mirroring techniques during the interviews. This allowed the members to ask follow-up questions in the interviewees’ language to gain better insights and acquire knowledge of the interviewees’ world views instead of the researchers’ world views. In addition, our interview guide was structured in such a way that initially open, and general questions were asked and then continued with specific questions.
**Guideline 6: Flexibility**
The interviews were conducted as semi-structured interviews. This allowed the team of interviewers to respond spontaneously to answers, react flexibly to the interviewee’s attitude, and follow up on interesting answers.
**Guideline 7: Ethics of Interviewing**
Interviewees were informed of the scope and duration before the interviews. Permission was obtained from all interviewees and repeated and taped before the interviews. All interviewees were informed that participation was voluntary and that their answers and data would be accessible only to the team of interviewers. Names and sensitive data have been pseudonymised after the interview, and they have been published anonymously. A summary of the results was delivered to the interviewees upon completion of the master’s theses. Since there is a lot of free work on social media platforms, we compensated the interviewees with vouchers as a thank-you gift. Some rejected the vouchers or preferred to donate to a non-profit organisation of their choice.

Appendix E: Interview guide for semi-structured interviews

**Briefing**
Thank you very much for taking the time for this interview. As part of my research project at the [name of university], I would like to find out how content creators stabilise their income in a dynamic environment like social media and what efforts content creators make to achieve this.<br>Before we start, I would like to point out that all collected data will be used anonymously as part of the research, and no conclusions can be drawn about individuals or companies. Only the research team will have access to the data. Participation in the interview is voluntary.
**Section 1: History & Motivation (10%)**
I would like to understand how you became a content creator. Please tell me what motivated you and what you did to become a content creator. Feel free to start from your beginnings. What does your job as a content creator look like? Can you describe a daily routine or weekly schedule for me?
**Section 2: Status & Livelihood (15%)**
What role does being a content creator play in your life? How do you make a living? Do you derive any income from your content creation activities?
**Section 3: Commitment (10%)**
What platforms do you use? What is the importance of YouTube for you? What is the importance of the above platforms for you?
**Section 4: Fluctuations (65%)**
Have you noticed fluctuations in your YouTube income? How do you feel about this fluctuation? Do you do anything about these fluctuations? If yes, what? Are there other triggers that drive your actions? What guides your content creation? Have your activities resulted in your income being more regular than before? You talked about activities X, Y & Z. What worked well?

**Parting**
We are at the end of the interview. Is there anything else you would like to share or comment on?<br><br>Can you point me to other content creators who also face these fluctuations (and whom I might contact if necessary)?<br><br>Thank you for your willingness and participation.
